# Supplementary material for: MOF-Derived Co Nanoparticles Catalyst Assisted by F- and N-Doped Carbon Quantum Dots for Oxygen Reduction
Source: Nanomaterials (Basel). 2023 Jul 18;13(14):2093. doi: 10.3390/nano13142093 (PMC10384604; doi:10.3390/nano13142093)
Supplement: Supplementary file 1 [file nanomaterials-13-02093-s001.zip › nanomaterials-2438378-supplementary.pdf]

Supporting information

MOF-derived Co nanoparticles catalyst assisted by F- and N-doped carbon quantum dots for oxygen reduction

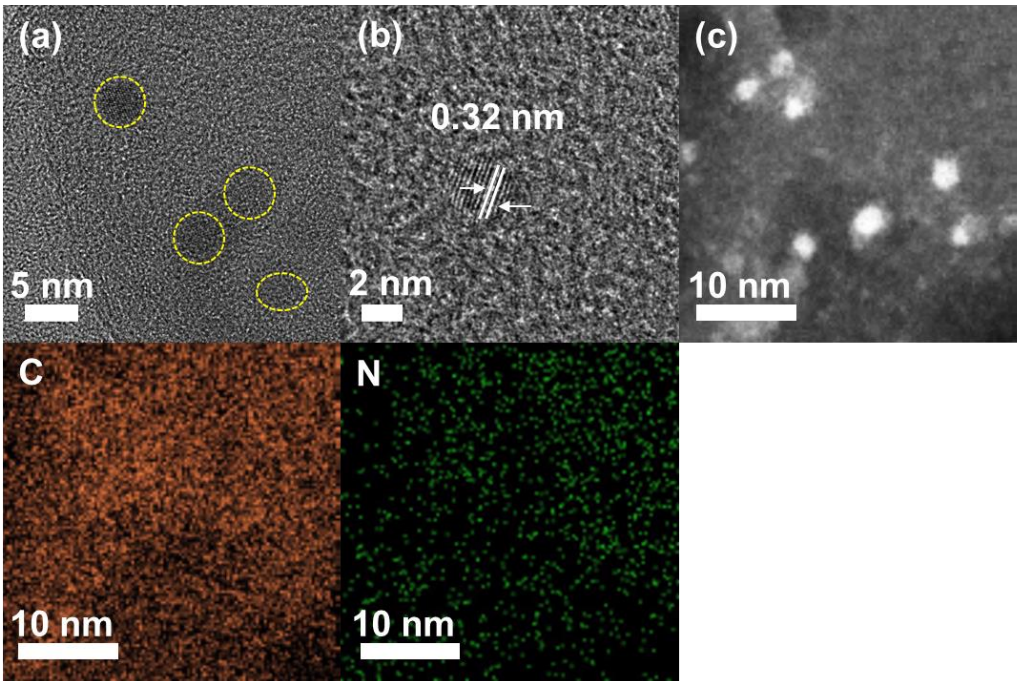

Figure S1. (a) Low- and (b) high-resolution TEM images, (c), EDS mapping results of NCQD.

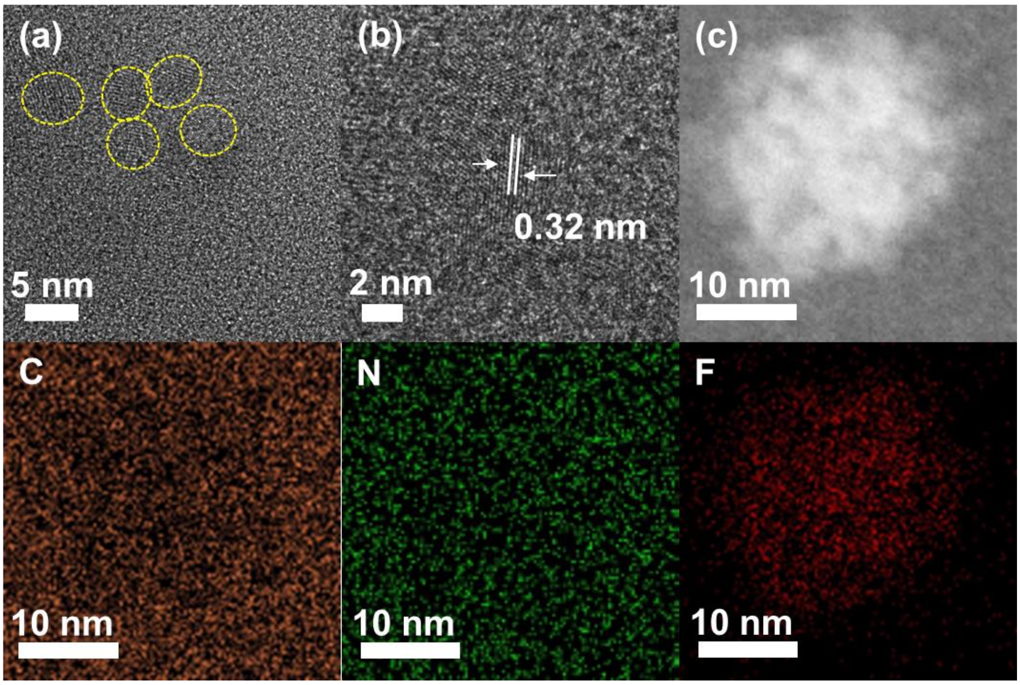

Figure S2. (a) Low- and (b) high-resolution TEM images, (c), EDS mapping results of FNCQD.

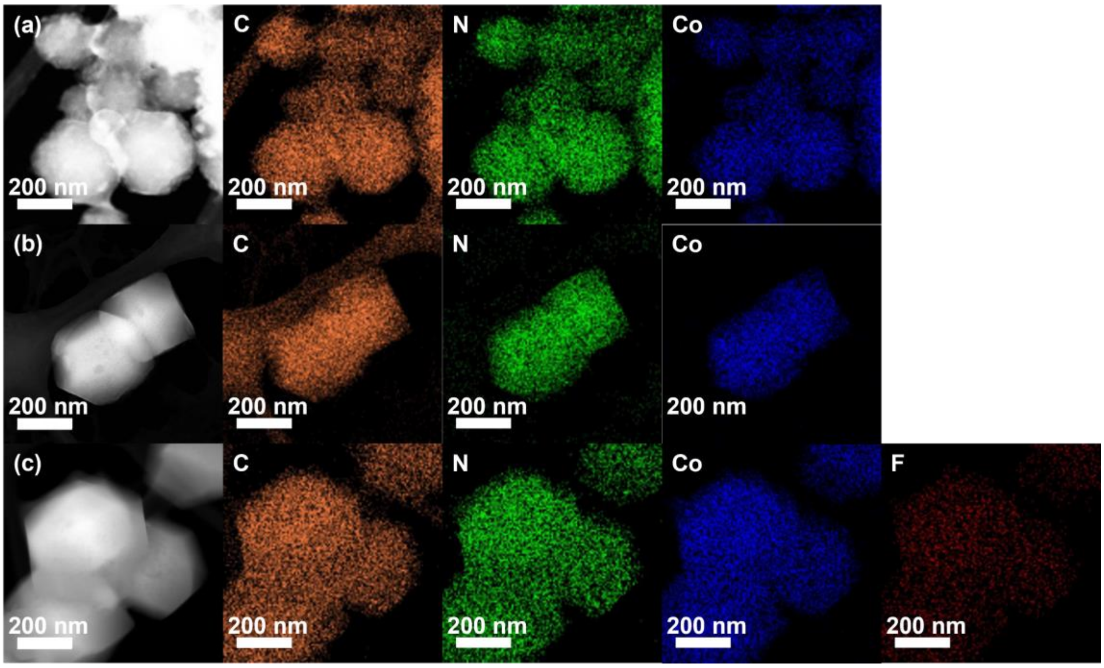

Figure S3. TEM- EDS mapping results of (a) ZIF-67, (b) NCQD/ZIF-67, and (c) FNCQD/ZIF-67.

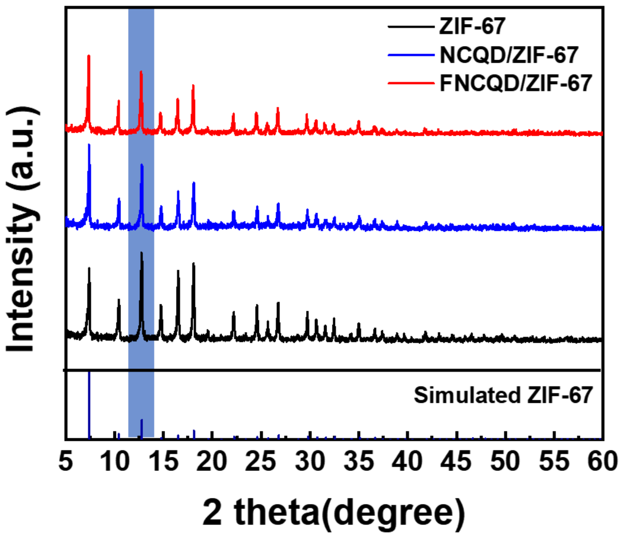

Figure S4. XRD patterns of ZIF-67, NCQD/ZIF-67 and FNCQD/ZIF-67.

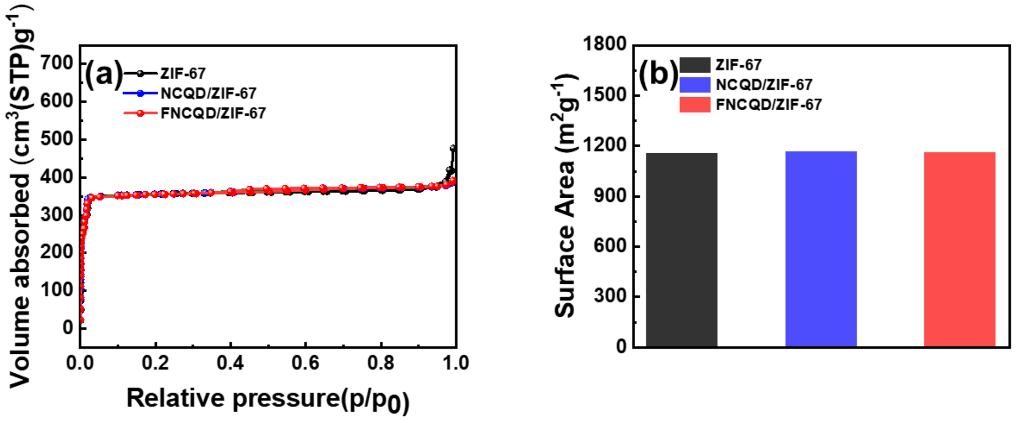

Figure S5. (a) BET gas adsorption plots, (b) change on BET of ZIF-67, NCQD/ZIF-67 and FNCQD/ZIF-67.

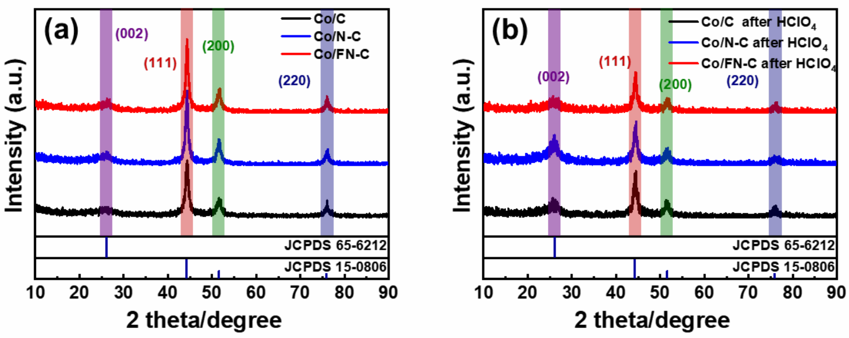

Figure S6. X-ray patterns of (a) before and (b) after $HClO_4$ treatment of Co/C, Co/N-C, and

Co/FN-C.

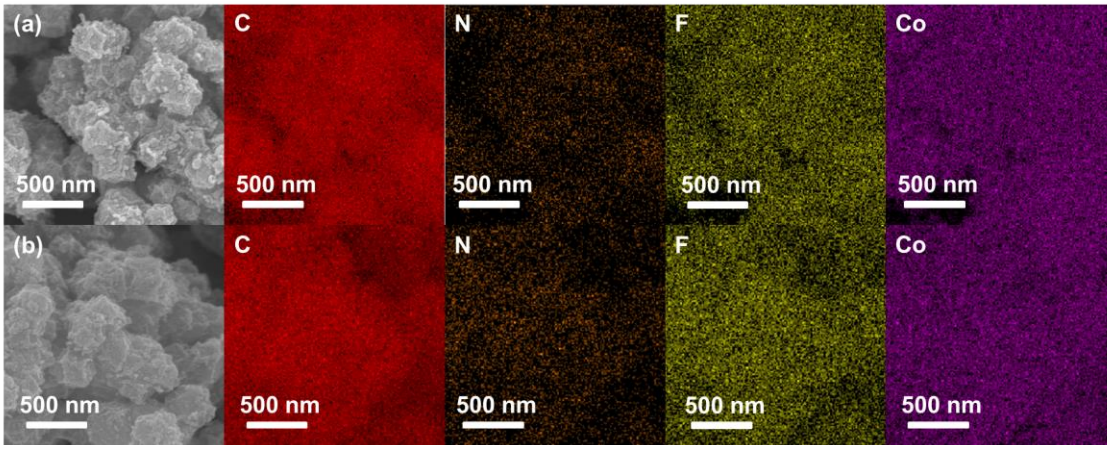

Figure S7. SEM-EDS mapping of C, N, F, and Co of (a) before and (b) after electrochemical test of Co/FN-C electrode.

Table S1. The chemical composition (atomic percent) of before and after the electrochemical test of Co/FN-C electrode.

Element	Before (atom %)	After (atom %)
C	62.80	71.29
N	15.77	9.68
O	12.88	11.58
F	0.21	0.21
Co	8.34	7.24
Total	100	100
